# A Cross-sectional Survey to Assess Awareness of Syndromic Surveillance by Clinicians Practicing Emergency Medicine: An Opportunity for Education and Collaboration

**DOI:** 10.5811/westjem.58392

**Published:** 2023-05-03

**Authors:** Gabriel Ann Haas, Brittany Hudson-Walsh, Adrienne Malik, Kellie Wark

**Affiliations:** * Kansas Department of Health and Environment, Kansas Syndromic Surveillance Program, Topeka, Kansas; † University of Kansas School of Medicine, Department of Emergency Medicine, Kansas City, Kansas; ‡ University of Kansas School of Medicine, Division of Infectious Disease, Kansas City, Kansas

## Abstract

**Introduction:**

Syndromic surveillance (SyS) is an important public health tool using de-identified healthcare discharge data from emergency department (ED) and urgent care settings to rapidly identify new health threats and provide insight into current community well-being. While SyS is directly fed by clinical documentation such as chief complaint or discharge diagnosis, the degree to which clinicians are aware their documentation directly influences public health investigations is unknown. The primary objective of this study was to evaluate the degree to which clinicians practicing in Kansas EDs or urgent care settings were aware that certain de-identified aspects of their documentation are used in public health surveillance and to identify barriers to improved data representation.

**Methods:**

We distributed an anonymous survey August–November 2021 to clinicians practicing at least part time in emergency or urgent care settings in Kansas. We then compared responses from emergency medicine (EM)-trained physicians to non-EM trained physicians. Descriptive statistics were used for analysis.

**Results:**

A total of 189 respondents across 41 Kansas counties responded to the survey. Of those surveyed, 132 (83%) were unaware of SyS. Knowledge did not differ significantly by specialty, practice setting, urban region, age, nor by experience level. Respondents were unaware of which aspects of their documentation were visible to public health entities, or how quickly records were retrievable. When asked about improving documentation for SyS, lack of clinician awareness (71.5%) was perceived as a greater barrier than electronic health record platform usability or time available to document (61% and 59%, respectively).

**Conclusion:**

This survey suggests that most practitioners in EM have not heard of SyS and are unaware of the invaluable role certain aspects of their documentation play in public health. Critical information that would be captured and coded into a key syndrome is often missing, but clinicians are unaware of what types of information may be most useful in their documentation, and where to document that information. Lack of knowledge or awareness was identified by clinicians as the single greatest barrier to enhancing surveillance data quality. Increased awareness of this important tool may lead to enhanced utility for timely and impactful surveillance through improved data quality and collaboration between EM practitioners and public health.

## INTRODUCTION

Syndromic surveillance (SyS) is a data collection strategy that informs public health about concerning trends in near real-time by analyzing patient-reported symptoms and electronic health record (EHR) documentation from clinicians in emergency departments (ED) and urgent care.^1^,^2^ The timely information that SyS can provide about current community well-being, and the ability to query free-text fields (eg, chief complaint, triage notes) in addition to discharge diagnosis, allow for early outbreak detection and active surveillance of a wide variety of public health indicators. Health departments work with hospitals to send de-identified visit data in batches as frequently as every hour, and the data is monitored on a daily basis to alert epidemiologists to potential health-related concerns.

Epidemiologists actively use SyS tools in their day-to-day practice, and there is great opportunity for collaboration with frontline clinicians providing the data input. For example, e-cigarette or vaping-associated lung injury, was initially identified when astute healthcare clinicians alerted public health practitioners to cases of respiratory failure among young adults, prompting widespread SyS queries to further quantify this public health problem and identify cases for investigation.^3^ More recently, SyS was used to assess the real-time impact of physical and social distancing rules implemented in the initial phase of the coronavirus coronavirus 2019 pandemic.^4^

Syndromic surveillance has also been used to prove the efficacy of vaccination initiatives by demonstrating a decrease in patients presenting to EDs for target diseases.^6^,^7^ Furthermore, SyS has been used to analyze extreme weather events, providing information to assist in statewide response plans.^8^–^10^ The public health applications of SyS are as vast as the data SyS obtains from EHR-documented symptoms and diagnoses, and the data can be used to more rapidly respond to emerging health threats than traditional sources of public health information.

As SyS systems use data generated from clinician documentation, the strength of the data collected is reliant on clinician awareness of the role of their documentation in SyS.^11^ To date, this relationship has not been well examined in public health or medical literature, which is surprising as SyS systems are fed directly by EHR documentation from acute care, urgent care, and ED settings (which in this manuscript we will consider collectively as “ED settings”). To better explore clinician understanding of SyS, we created a survey evaluating their awareness of SyS and perception of EHR data-collection methods. We hypothesized that emergency clinicians in our state are largely unaware of SyS and unaware of the invaluable role their documentation plays in the aggregation of data for public health action. Given that Kansas has a robust SyS system and is leading the way in SyS outreach and application, the state was well situated for an investigation of this hypothesis.

## METHODS

We conducted a cross-sectional survey designed to assess emergency clinician (physician and midlevel practitioner) awareness and understanding of SyS through an electronic survey questionnaire. Twelve of 29 survey questions gathered respondent demographics, training level, primary practice setting, and assessed their understanding of SyS and perceived barriers toward improving documentation for public health purposes. (For full survey template see supplement.) The questionnaire was created by the Kansas Syndromic Surveillance Program. The survey instrument was piloted with nine physicians and underwent three revisions.

**Population Health Research Capsule**

What do we already know about this issue?

*Syndromic surveillance (SyS) is a public health tool using de-identified ED visit records to rapidly assess current health threats*.

What was the research question?

*Are clinicians in emergency medicine aware their documentation is important for public health surveillance?*

What was the major finding of the study?

*Of 189 clinicians surveyed, 83% were unfamiliar with SyS or the role their charting plays in public health*.

How does this improve population health?

*Increasing awareness of SyS within emergency medicine will inform public health practice through collaboration to target surveillance and enhance data quality*.

Survey subjects were eligible to participate if they identified as practicing in an EM or urgent care (UC) setting in the state of Kansas (eg, EM-trained, and non-urban family medicine [FM], internal medicine [IM] clinicians and rural physician assistants [PA]). In rural counties, the ED did not have to be the primary practice setting provided the clinician identified as practicing in the ED at least part time. We acquired clinicians’ emails from the Kansas Board of Healing Arts database, and we contacted potential survey participants via email correspondence and the Kansas Chapter of the American College of Emergency Physicians (KS ACEP). Responses were anonymous. The survey was disseminated and stored with survey software (Qualtrics XM, Provo, UT) from August 1–November 12, 2021, and participants were contacted multiple times. We analyzed qualitative data using survey analytic descriptive statistics (SAS Institute, Inc, Cary, NC). Awareness and perception differences were compared with Pearson chi-square tests.

## RESULTS

Of 1,553 EM, FM, IM physicians and PAs queried, 189 responded. Of those queried, 480 were formally trained in EM. There is no existing source to quantify how many clinicians practice in Kansas EDs. Further, not all physicians queried may have been eligible to participate in the survey as outlined by our communication. The response rate for emergency physicians at our state’s large academic medical facility reached 38%. Responses were received from clinicians in 41 counties, reflecting excellent Kansas clinician representation given that three-quarters of the state population resides in just six counties.

See Table 1 for responses by practice setting, age range, and level of training. The majority of respondents identified as EM-specialized (46.7%) followed by FM (24.5%) and IM (20.7%). Primary practice setting was identified as the ED in 48.1%, followed by “other” in 32.2%, inpatient for 15.3%, and 4.4% urgent care. As Kansas is a largely rural state, emergency clinicians in critical access areas are often physicians or mid-level practitioners from a variety of specialty-training backgrounds, practicing acute care primarily in non-traditional EM settings.

The majority of survey respondents indicated they were unfamiliar with SyS, and the role that EHR documentation serves in public health. When discussing public health and SyS, 75% of respondents indicated “no” when asked “Have you heard of a subset of public health surveillance called syndromic surveillance?” Only 17% of respondents indicated they had heard of SyS, although none indicated where they had previously learned of SyS. Awareness of SyS did not significantly differ by practice setting, academic vs non-academic center, age, nor by clinician training (Table 1). For the analysis, we compared the relative difference in responses between EM and non-EM trained physicians and found no significant differences between the responses.

Respondents were unsure which aspects of documentation are visible to public health, how quickly data is received, and what conditions are monitored using SyS (Table 2). When asked what their perceived barriers were to improving clinician documentation as it relates to public health data, the most popular three answers were clinician lack of awareness (most frequently chosen), electronic health systems (second most frequent response), and time (third most frequent response). (These answer choices do not reflect accurate information related to SyS data collection in Kansas.)

## DISCUSSION

The data obtained in this survey supports our hypothesis that emergency physicians and other clinicians who practice in ED settings are unfamiliar with SyS. Respondents were also unclear about the role EHRs serve in capturing public health trends using SyS. Although not all clinicians identified as practicing primarily in an ED setting, the distribution of responses was similar to a 2020 study demonstrating that FM physicians represented nearly half of the overall physician workforce.^12^ Additionally, we found that awareness did not differ significantly by primary practice setting or formal training. This near ubiquitous lack of awareness was identified by clinicians as the largest barrier to improving EHR documentation for SyS, ahead of constraints of EHR platforms and the time available to document thoroughly. While there is minimal ability to broadly impact the types of EHR systems used, or the time available for clinicians to document patient encounters, increasing awareness among ED practitioners about SyS is a feasible intervention that could impact the future of SyS practice.

This survey fills a gap in the literature addressing the understanding of SyS by clinicians. Our survey results indicate clinicians are unsure what types of information might be useful and where in the EHR documentation. They are not sure what types of conditions and social determinants of health epidemiologists are attempting to monitor. When asked about what this data is used for, respondents were more likely to select that public health monitors reportable infectious diseases or conditions only via state mandate of importance. In reality, public health is using SyS data to monitor a wide variety of health outcomes.^13^ Its use has recently been expanded beyond outbreak detection for real-time monitoring of a wide variety of conditions including mental health-related visits, drug overdose, environmental health impacts, and surveillance of patterns in trauma, violence, and injury.^8^,^10^,^14^,^15^,^16^ Public health can do more to actively inform emergency clinicians about conditions and codes of interest or work directly with them to actively monitor conditions of concern.^13^

From direct conversations with the National Syndromic Surveillance Program and ACEP we suspect awareness of SyS is low nationally, not just in Kansas. In fact, Kansas has been one of the state programs leading the way in SyS outreach and application. Increasing awareness of SyS by clinicians has the potential to unearth many meaningful applications for this data through academic public health partnerships and applied public health research. Physicians in Kansas changed the way they document to include additional contextual diagnosis codes not included prior to knowledge about SyS. Codes or language of interest may be determined in collaboration with local public health agencies for emerging health threats or community events. This is also an opportunity to enhance the feedback loop between public health and medicine to target surveillance efforts and provide useful data back to clinicians. Improving the quality of SyS data at the clinician level through increased awareness has obvious implications for future advances in the way we predict, monitor, and respond to disease on a local and national level.

## LIMITATIONS

Although our overall response rate was typical for e-mail-based survey studies of clinicians without incentives, our study is limited by the number of respondents. While our responses are representative of a wide variety of practice settings and experience levels, we cannot exclude the possibility of non-response bias or bias from the survey instrument itself. The length of the survey was likely a factor, as not all respondents answered every question. Additionally, while we suspect our results are likely generalizable to other states, the survey in this study was only administered to practitioners in Kansas. Many of our responses came from clinicians who are not formally EM trained or may be practicing in ED settings part time. While this could generate concerns about reaching our intended audience, it is also a strength of our study because it demonstrates that we captured responses from non-traditional, rural clinicians who practice in ED settings. Finally, the high response rate of academic practitioners in EM to the survey may introduce bias that makes the results less representative of the statewide ED workforce.

## CONCLUSION

Frontline clinicians practicing in ED settings in the state of Kansas are largely unaware of syndromic surveillance and the critical role their documentation plays within this facet of the public health system. Clinicians reported that a lack of understanding of SyS is a significant barrier to making changes to electronic health record-level documentation that would improve the quality of data collected for SyS. These findings represent an opportunity to increase education and collaboration between EM and public health for surveillance purposes.

## Supplementary Information



## Figures and Tables

**Table 1:** Respondent breakdown and calculated P-values to assess whether awareness of syndromic surveillance differed significantly by hospital type, age, role, specialty, or practice setting.

Demographics	All respondents (N=189)	Have you heard of syndromic surveillance?*			

	% (n)	Yes, % (n)	No, % (n)	Unsure, % (n)	Group P-value (chi-square)
		17.0% (27/159)	74.8% (119/159)	8.2% (13/159)	
Hospital type					
Critical access hospital	14.0% (23/164)	0% (0/21)	90.5% (19/21)	9.5% (2/21)	0.152
Teaching facility	37.8% (62/164)	20.8% (11/53)	71.7% (38/53)	7.5% (4/53)	
Non-teaching facility	25.6% (42/164)	16.7% (6/36)	80.6% (29/36)	2.8% (1/36)	
Other (Urgent care, ambulatory)	22.6% (37/164)	21.9% (7/32)	62.5% (20/32)	15.6% (5/32)	
Age					
20–29	15.4% (29/188)	0% (0/25)	84% (21/25)	16% (4/25)	0.173
30–49	54.8% (103/188)	20.5% (17/83)	73.5% (61/183)	6.0% (5/83)	
50–69	25.5% (48/188)	20.9% (9/43)	69.8% (30/43)	9.3% (4/43)	
70+	4.3% (8/188)	12.5% (1/8)	87.5% (⅞)	0% (0/8)	
Level of training					
Resident or fellow	18.6% (34/183)	14.8% (4/27)	74.1% (20/27)	11.1% (3/27)	0.468
Attending	67.8% (124/183)	18.9% (21/111)	73.0% (81/111)	8.1% (9/111)	
Mid-level practitioner	13.7% (25/183)	9.5% (2/21)	85.7% (18/21)	4.8% (1/21)	
Practice location					
Urban or semi-urban	75.5% (143/189)	20% (23/115)	72.2% (83/115)	7.8% (9/115)	0.347
Rural	24.3% (46/189)	10% (4/40)	80% (32/40)	10% (4/40)	
Primary practice setting					
Emergency department	48.1% (88/183)	16.9% (13/77)	76.6% (59/77)	6.5% (5/77)	0.676
Inpatient	15.3% (28/183)	14.3% (3/21)	76.2% (16/21)	9.5% (2/21)	
Urgent care	4.4% (8/183)	37.5% (3/8)	62.5% (5/8)	0% (0/8)	
Other (clinic, tele-medicine)	32.2% (59/183)	15.1% (8/53)	73.6% (39/53)	11.3% (6/53)	
Specialty					
Emergency medicine	46.7% (86/184)	21.3% (16/75)	73.3% (55/75)	5.3% (4/75)	.806
Family medicine	24.5% (45/184)	16.7% (7/42)	71.4% (30/42)	11.9% (5/42)	
Internal medicine	20.7% (38/184)	10.3% (3/29)	79.3% (23/29)	10.3% (3/29)	
Pediatrics	3.3% (6/184)	16.7% (1/6)	66.7% (4/6)	16.7% (1/6)	
Other (hematology, oncology, occupational medicine, endocrinology, geriatrics, toxicology)	4.9% (9/184)	28.6% (2/7)	57.1% (4/7)	14.3% (1/7)	

* Not all respondents answered every question. Total responses to this question may vary from imputed practitioner information.

*SyS*, syndromic surveillance

**Table 2:** All analyzed survey questions and their results.

Awareness	EM Respondents, % (n)	All Respondents, % (n)
Have you heard of syndromic surveillance?		
yes	21.3% (16/75)	17.0% (27/159)
no	73.3% (55/75)	74.8% (119/159)
unsure	5.3% (4/75)	8.2% (13/159)
Is public health able to monitor de-identified healthcare discharge data for surveillance purposes?		
yes	36.0% (27/75)	30.2% (48/159)
no^**^	4.0% (3/75)	6.3% (10/159)
unsure	60.0% (45/75)	53.4% (101/159)
Which aspects of documentation can be monitored for public health surveillance? (Select all that apply.)		
unsure	63.1% (41/64)	65.4% (85/130)
ICD diagnosis codes	51.6% (33/64)	50.8% (66/130)
patient demographics (e.g. age, county)	20.3% (13/64)	23.1% (30/130)
procedure codes	15.6% (10/64)	16.9% (22/130)
chief complaint	14.1% (9/64)	13.9% (18/130)
identifiable patient data (e.g. name, address)^**^	6.3% (4/64)	26.9% (35/130)
vital signs	4.7% (3/64)	5.4% (7/130)
triage notes	4.7% (3/64)	4.6% (6/130)
Clinician assessments (e.g. HPI, assessment, and plans)^**^	0.0% (0/64)	10.8% (14/130)
When ED or UC surveillance is possible, how soon is it generally retrievable after ED discharge?		
Unsure	78.1% (50/64)	80.8% (105/130)
1–12 hours	7.8% (5/64)	4.6% (6/130)
12–48 hours	6.3% (5/64)	10.0% (13/130)
2–7 days^**^	0% (0/64)	4.6% (6/130)
1–2 weeks^**^	4.7% (3/64)	3.1% (4/130)
Not possible^**^	3.1% (2/64)	1.6% (2/130)
Which data is monitored from ED/urgent care EHR systems at the public health level? (Select all that apply.)		
Unsure	56.3% (36/64)	56.2% (73/130)
Reportable infectious diseases	34.3% (22/64)	36.2% (47/130)
Critical diseases only by state mandate of importance	34.3% (22/64)	34.6% (45/130)
Emerging conditions of interest (e.g. EVALI)	29.7% (19/64)	29.2% (38/130)
Environmental exposures (e.g. weather related)	28.2% (18/64)	28.5% (37/130)
Visits following a mass gathering or disaster	26.6% (17/64)	28.5% (37/130)
Adverse events (e.g. vaccine side effects)	25.0% (16/64)	26.2% (34/130)
Trauma-related (e.g. child abuse, interpersonal violence)	25.0% (16/64)	25.4% (33/130)
Syndromes (e.g. diarrhea, rash + fever)	15.6% (10/64)	18.5% (24/130)
Acute conditions (e.g. AMI, appendicitis)	12.5% (8/64)	16.9% (22/130)
Mental health-related visits	18.8% (12/64)	15.4% (20/130)
What barriers would you perceive as most affecting your ability to improve documentation for public health surveillance data? (Select your top 3.)		
Clinician lack of awareness (e.g. clinicians do not realize certain documentation is monitored or important for surveillance)	49/62 (79.0%)	71.5% (93/130)
Electronic health systems (i.e. usability, platforms, and vendors)	66.1% (41/62)	60.8% (79/130)
Time required to document	64.5% (40/62)	59.2% (77/130)
Perceived level of importance (e.g. irrelevance of patient history to coding)	50.0% (31/62)	43.1% (56/130)
Lack of standardization/proper codes	40.3% (25/62)	39.2% (51/130)
Lack of collaboration between medicine and public health	35.5% (22/62)	36.9% (48/130)
Nurse or receptionist lack of awareness (e.g. documentation of CC or triage-note data by nurse or receptionist is not perceived as important)	33.9% (21/62)	32.3% (42/13)

*ICD*, International Classification of Diseases; *HPI*, history of present illness; *EVALI*, e-cigarette or vaping product use-associated lung injury; *AMI*, acute myocardial infarction; *CC*, chief complaint; *ED*, emergency department; *EHR*, electronic health record.

## References

[1] Colón-González FJ, Lake IR, Morbey RA (2018). A methodological framework for the evaluation of syndromic surveillance systems: a case study of England. BMC Public Health.

[2] Yoon PW, Ising AI, Gunn JE (2017). Using syndromic surveillance for all-hazards public health surveillance: successes, challenges, and the future. Public Health Rep.

[3] Krishnasamy VP, Hallowell BD, Ko JY (2020). Update: characteristics of a nationwide outbreak of e-cigarette, or vaping, product use - associated lung injury - United States, August 2019 to January 2020. MMWR Morb Mortal Wkly Rep.

[4] Hartnett KP, Kite-Powell A, DeVies J (2020). National Syndromic Surveillance Program community of practice. impact of the COVID-19 pandemic on emergency department visits - United States, January 1, 2019-May 30, 2020. MMWR Morb Mortal Wkly Rep.

[5] Bellazzini MA, Minor KD (2011). ED syndromic surveillance for novel H1N1 spring 2009. Am J Emerg Med.

[6] Hughes HE, Elliot AJ, Hughes TC (2020). Using emergency department syndromic surveillance to investigate the impact of a national vaccination program: a retrospective observational study. PLoS One.

[7] Christie A, Henley SJ, Mattocks L (2021). Decreases in COVID-19 cases, emergency department visits, hospital admissions, and deaths among older adults following the introduction of COVID-19 vaccine — United States, September 6, 2020–May 1, 2021. MMWR Morb Mortal Wkly Rep.

[8] Dirmyer VF (2018). Using real-time syndromic surveillance to analyze the impact of a cold weather event in New Mexico. J Environ Public Health.

[9] Tsai S, Hamby T, Chu A (2016). Development and application of syndromic surveillance for severe weather events following Hurricane Sandy. Disaster Med Public Health Prep.

[10] Schramm PJ, Vaidyanathan A, Radhakrishnan L (2021). Heat-related emergency department visits during the Northwestern heat wave — United States, June 2021. MMWR Morb Mortal Wkly Rep.

[11] Smith GE, Elliot AJ, Lake I (2019). Syndromic surveillance: two decades experience of sustainable systems: It’s people, not just data!. Epidemiol Infect.

[12] Bennett CL, Gerard WA, Cullen JS (2021). National study on the contribution of family physicians to the US emergency physician workforce in 2020. J Am Board Fam Med.

[13] Mandl KD, Overhage JM, Wagner MM (2004). Implementing syndromic surveillance: a practical guide informed by the early experience. J Am Med Inform Assoc.

[14] Slavova S, Rock P, Bush HM (2020). Signal of increased opioid overdose during COVID-19 from emergency medical services data. Drug Alcohol Depend.

[15] Zwald ML, Holland KM, Bowen DA (2022). Using the Centers for Disease Control and Prevention’s National Syndromic Surveillance Program data to monitor trends in US emergency department visits for firearm injuries, 2018 to 2019. Ann Emerg Med.

[16] Smith GE, Harcourt SE, Hoang U (2022). Mental health presentations across health care settings during the first 9 months of the COVID-19 pandemic in England: retrospective observational study. JMIR Public Health Surveill.

