# Supplementary material for: A Cross-sectional Survey to Assess Awareness of Syndromic Surveillance by Clinicians Practicing Emergency Medicine: An Opportunity for Education and Collaboration
Source: West J Emerg Med. 2023 May 3;24(3):424–30. doi: 10.5811/westjem.58392 (PMC10284521; doi:10.5811/westjem.58392)
Supplement: Supplementary file 1 [file wjem-24-424-supplement.docx]

Syndromic Surveillance Provider Awareness Survey

Start of Block: Background Information

Q1 Which county in Kansas do you **primarily** work?

▼ I do not work in KS (1) ... Wyandotte (106)

Q2 What is your age range?

- 20-29 (1)
- 30-49 (2)
- 50-69 (3)
- 70+ (4)

Q3 Are you a physician?

- Yes (1)
- No (2)

Display This Question:

If Are you a physician? = Yes

Q4 If you are a physician, what is your level of training?

- Attending physician (1)
- Resident or fellow (2)

Display This Question:

If Are you a physician? = Yes

Q5 What is your specialty? (select all that apply)

- Emergency medicine (1)
- Family medicine (2)
- Internal medicine - hospitalist (3)
- Internal medicine - outpatient (4)
- Pediatrics (5)
- Surgery - trauma (6)
- Surgery - general (7)
- Other subspecialty: (8) ________________________________________________

Display This Question:

If Are you a physician? = No

Q6  If you are a non-physician provider, what is your role?

- APRN (1)
- PA (2)
- Student (medical, PA or APRN) (3)
- Other (4) ________________________________________________

Q7 Practice setting (predominantly)

- ED (1)
- Urgent care (2)
- Inpatient hospital setting (3)
- Other (4) ________________________________________________

Q8 What type of ED or urgent care do you work in?

- Critical access facility (1)
- Non-teaching facility (2)
- Teaching facility (3)
- Other (4) ________________________________________________

Q9 How long have you worked in your current role (in years)?

- 0-5 (1)
- 6-10 (2)
- 11-20 (3)
- 21+ (4)

End of Block: Background Information

Start of Block: Knowledge of public health and syndromic surveillance

Q10 What are the main purposes of public health surveillance (select all that apply)

- Unsure (1)
- Monitor trends in disease over time (2)
- Prevent and control disease (3)
- Detect and identify disease outbreaks (4)
- Determine the prevalence of disease (5)
- Health education and disease advocacy (6)
- Other (7) ________________________________________________

Q11 Have you heard of a subset of public health surveillance called **syndromic surveillance**?

- Unsure (1)
- Yes (2)
- No (3)

Q12 Is the health department able to monitor de-identified ED or urgent care visit records for public health surveillance purposes?

- Unsure (1)
- Yes (2)
- No (3)

Q13 What can **syndromic surveillance** be used for (select all that apply)

- Unsure (1)
- Alternative and/or more rapid methods to report diseases (2)
- Detect outbreaks (3)
- Early detection of emerging threats (e.g., EVALI [vaping lung injury], opioid overdoses) (4)
- Health education and disease advocacy (5)
- Monitor facilitie's EHRs identifiable patient data for contact tracing in outbreak response (6)
- Monitor non-infectious events (e.g., injuries, suicides) (7)
- Monitor trends in diseases over time (8)
- Prevent diseases (9)
- Reporting system after diagnosis has been made (10)
- Report labs rapidly to public health (e.g., positive HIV signals an alert) (11)
- Report targeted events by certain geographic region (e.g., selected sites monitor and report positive nasopharyngeal flu swabs) (12)
- Use existing health data (via EHR) for immediate analysis and feedback (13)
- Use real time data to identify potential threats (14)
- Other (15) ________________________________________________

End of Block: Knowledge of public health and syndromic surveillance

Start of Block: Syndromic surveillance awareness

Q14 When ED (and urgent care) syndromic surveillance monitoring is possible, how soon is it generally retrievable after ED discharge?

- Unsure (1)
- 1-12 hours (2)
- 12-48 hours (3)
- 2-7 days (4)
- 1-2 weeks (5)
- It is not currently possible (6)

Q15 Which components of an ED or urgent care visit are possible to monitor at the public health level (select all that apply)

- Unsure (1)
- De-identified patient visit records (2)
- Identifiable patient data (e.g., name, address) (3)
- Diagnosis codes (4)
- Free-text fields (e.g., chief complaint, admit reason, triage notes) (5)
- Assessments by provider (e.g., HPI, assessment and plans) (6)

Q16 Which data is monitored from ED/urgent care EHR systems at the public health level (select all that apply)

- Unsure (1)
- Acute conditions (e.g., AMI, appendicitis) (2)
- Adverse events (e.g., vaccine side effects) (3)
- Critical diseases only by state of mandate importance (e.g., COVID-19, anthrax) (4)
- Emerging conditions of interest (e.g., EVALI) (5)
- Environmental exposures (e.g. heat waves, carbon monoxide) (6)
- Following a mass event or gathering (e.g., Super Bowl and assault or injuries) (7)
- Following a disaster (e.g., chemical release, explosions) (8)
- Injuries (by type, mechanism, and circumstance) (9)
- Mental health related visits (10)
- Preventative and circumstantial visits (e.g., med refills, hypertensive visits) (11)
- Provider to provider documentation (12)
- Syndromes (e.g., diarrhea, rash+fever) (13)
- Specific infectious diseases and reportable conditions (14)
- Trauma-related (e.g., child abuse, rape) (15)
- Other: (16) ________________________________________________

Q17 Which of the following data is monitored from ED/urgent care EHR at the state level (select all that apply)

- Unsure (1)
- Chief complaint (2)
- Data and time of visit (3)
- Discharge disposition (4)
- Health status (e.g., pregnant, diabetic) (5)
- Initial impression in ED, admit reason (6)
- Non-identifying patient demographics (e.g., age, county) (7)
- Patient identifying data (e.g., name, address) (8)
- Travel history (9)
- Triage notes (10)
- Vital signs (11)
- Procedure codes (12)
- Diagnosis codes (13)
- Other (14) ________________________________________________

Q18 How often do you document clinical context (e.g., mechanism of injury, travel history, prompting incident or special population status) in the chief complaint or reason for visit?

- A nurse or receptionist does this (1)
- Never (2)
- Rarely (3)
- Sometimes (4)
- Frequently (5)
- Always (6)

Q19 How familiar are you with ICD-10 coding structure and coverage?

- Not familiar at all (1)
- Slightly familiar (2)
- Moderately familiar (3)
- Very familiar (4)

Q20 How often do you use the following types of codes?

|  | Frequency of use | | | | |
| --- | --- | --- | --- | --- | --- |
|  | Frequently (1) | Sometimes (2) | Rarely (3) | Never (4) | I didn't know there were codes for this (5) |
| Activity or mechanism of Injury (e.g., cooking, heavy lifting, biking) (1) |  |  |  |  |  |
| Social determinants of health (e.g., unemployment, literacy level, residency status) (2) |  |  |  |  |  |
| Factors influencing health (e.g., nicotine dependence, pregnancy, obesity) (3) |  |  |  |  |  |
| Codes for Location (e.g., school, outdoors, mass gatherings) (4) |  |  |  |  |  |
| External Cause Codes: Environmental (snow/Ice, weather, occupational, life stressors) (5) |  |  |  |  |  |
| External Cause Codes: Medical care related (e.g., s/p procedure or surgery, medical device-related) (6) |  |  |  |  |  |

Q21 How much do the following factors influence your decision to include important information in the chief complaint, admit reason documentations or code selections?

|  |  | | | |
| --- | --- | --- | --- | --- |
|  | Strongly influences (1) | Sometimes influences (2) | Rarely influences (3) | Never influences (4) |
| Time (e.g., time to go back and edit original input) (1) |  |  |  |  |
| Awareness of the impact of documentation (e.g., did not feel clinically useful to go back and change/edit CC or codes) (2) |  |  |  |  |
| Relative importance (i.e., CC and discharge disposition are less important for clinical care) (3) |  |  |  |  |
| Administrative reporting requirements (e.g., must document disposition before able to leave the ED) (4) |  |  |  |  |
| Patient limitations (e.g., poor patient historians/details) (5) |  |  |  |  |

Q22 How important do you think it is for providers to be aware of trends in public health surveillance?

- Not important (1)
- Sometimes important (2)
- Frequently important (3)
- Always important (4)

Q23 How important do you think it is to improve data quality for public health surveillance?

- Not important (1)
- Slightly important (2)
- Moderately important (3)
- Very important (4)
- Critically important (5)

Q24 If you knew your daily documentation and coding impacted public health actions or improved time to outbreak detection, would you be more inclined to improve the most pertinent parts of your documentation?

- Yes (1)
- Maybe (2)
- No (3)

Q25 If you knew specific codes/language were more likely to trigger an event of concern (e.g., water exposure + dyspnea + diarrhea triggers harmful algal bloom alert) would you be more apt to use those codes and language of interest?

- Yes (1)
- Maybe (2)
- No (3)

Q26 What **barriers** do you perceive **most** affect your ability to improve your documentation related to public health surveillance data? (select the top 3)

- Electronic health systems (e.g., usability, platforms and vendors) (1)
- Lack of collaboration between medicine and public health (2)
- Lack of standardization/proper codes (3)
- Nurse or receptionist lack of awareness (e.g. documentation of CC or triage by nurse/receptionist-entered type of data is not perceived as important) (4)
- Perceived level of importance (e.g. irrelevance of patient history to coding) (5)
- Provider lack of awareness (e.g. providers do not realize certain documentation is monitored or important to public health surveillance) (6)
- Time (7)
- Other (8) ________________________________________________

Q26 Would it be helpful for you to see public health concerns of interest in your area, or to receive tailored alerts?

- Yes (1)
- Maybe (2)
- No (3)

Q27 Do you receive state or local health department alerts about public health emergencies or health-related topics of concern (e.g., KS health alert network [HAN] texts or emails)?

- Unsure (1)
- Yes (2)
- No (3)

Q28 If you are interested in more information on syndromic surveillance and/or how your documentation and coding can improve local or statewide efforts– in which ways would you prefer to receive this information or education (select all that apply)

- Not interested (1)
- Annual conferences (2)
- In-person didactics (grand grounds or conferences) (3)
- CDC or national website (4)
- Local health dept website (5)
- State health dept website (6)
- Listserv (7)
- Podcasts (8)
- Print publications / newsletters (9)
- Social media (10)
- Webinars (11)
- Work group (12)
- Other (13) ________________________________________________

Q29 And who would you prefer to receive this health surveillance type of information or education from (select all that apply)

- Not interested (1)
- Chief medical officer / medical staff (2)
- Program director (3)
- Administration (4)
- Local health department (5)
- State health department (6)
- National or state experts (7)
- Other (8) ________________________________________________

End of Block: Syndromic surveillance awareness
